# Deep Learning Based Vehicle Detection on Real and Synthetic Aerial Images: Training Data Composition and Statistical Influence Analysis

**DOI:** 10.3390/s23073769

**Published:** 2023-04-06

**Authors:** Michael Krump, Peter Stütz

**Affiliations:** Institute of Flight Systems, University of the Bundeswehr Munich, 85579 Neubiberg, Germany; peter.stuetz@unibw.de

**Keywords:** convolutional neural networks, deep learning, image descriptors, object detection, reality gap, synthetic training data, UAV, vehicle detection, virtual simulation, YOLOv3

## Abstract

The performance of deep learning based algorithms is significantly influenced by the quantity and quality of the available training and test datasets. Since data acquisition is complex and expensive, especially in the field of airborne sensor data evaluation, the use of virtual simulation environments for generating synthetic data are increasingly sought. In this article, the complete process chain is evaluated regarding the use of synthetic data based on vehicle detection. Among other things, content-equivalent real and synthetic aerial images are used in the process. This includes, in the first step, the learning of models with different training data configurations and the evaluation of the resulting detection performance. Subsequently, a statistical evaluation procedure based on a classification chain with image descriptors as features is used to identify important influencing factors in this respect. The resulting findings are finally incorporated into the synthetic training data generation and in the last step, it is investigated to what extent an increase of the detection performance is possible. The overall objective of the experiments is to derive design guidelines for the generation and use of synthetic data.

## 1. Introduction

Carrying sensor systems, UAVs (Unmanned Aerial Vehicles) are used for a wide variety of applications, such as area surveillance [1,2] and infrastructure inspection [3,4], land survey [5,6] or in the field of disaster control [7,8]. The advantages of such unmanned systems are a generally high flexibility and mobility [9], cost reduction, and their possibility to be used in safety-critical situations. In order to minimize the workload of the operators, a higher degree of automation of the UAV systems is especially aimed at multi-UAV applications or Manned-Unmanned Teaming concepts (MUM-T) [10,11,12]. This also concerns the integrated and real-time capable sensor data processing on board of the UAV [10,11,12]. In this context, the increased use of Artificial Intelligence (AI) methods can be observed [13,14,15,16,17]. However, the performance of such algorithms is highly influenced by the availability, quantity, and variance of suitable test and training datasets [18,19]. In the field of airborne sensing, their collection is only possible with complex and costly flight missions. Such data regularly only captures a fraction of the variance that subsequently occurs, is limited by legal restrictions, and requires manual labeling of the recorded data. Overall, this can result in algorithm models with low robustness to fluctuating environmental conditions [20].

One approach to circumvent these issues is to use virtual simulation environments to generate synthetic sensor data to augment existing real-world training data, increase their robustness, or replace them entirely. In addition, synthetic data generation offers further advantages in terms of cost reduction through shorter product development cycles, exact reproducibility of experiments, and automated generation of additional image information and highly accurate ground truth annotations. The goal is to investigate to what extent this synthetic data are suitable for use with Computer Vision (CV) algorithms. In this context, the term Reality Gap is used to describe the reason for differences in system behavior between the domains of simulation and reality [21,22,23].

### 1.1. Current State of Research

The literature already contains several sources that consider the use of virtual simulation environments in the field of CV algorithms. Many of them deal with the generation of photorealistic training datasets. In study [18], a fully annotated synthetic dataset for training the Faster RCNN network for vehicle detection is generated using a game engine and its performance is evaluated. Study [19] takes a similar approach for the semantic segmentation use case and shows that synthetic data augmentation can reduce the amount of expensive hand-annotated real data to one third with the same performance. Moreover, in study [24], real training data were augmented with synthetic image material and positive effects were recorded. In studies [25,26], a physics-based modeling and simulation environment for training and test data generation for deep learning based detectors was presented and used to optimize the training parameters. In the field of autonomous driving, the real KITTI dataset [27] serves as a benchmark for a variety of CV applications. Gaidon et al. [28] generated remodeled virtual duplicates (VKITTI) on this basis and used them for training multi-object tracking algorithms. They also showed that an evaluation of influencing parameters such as weather and lighting is possible in the simulation.

This approach to using synthetic data for testing purposes is also discussed in several sources. For example, in studies [29,30], in the area of ground-based vehicle detection, the effects of different methods of synthetic shadow generation and simulation of interference effects were evaluated. In study [31], the impact of different illumination conditions on the performance of common Convolutional Neural Networks (CNN) for object detection was investigated. The authors of [32] used synthetic data to investigate the susceptibility of CNNs to missing or incorrect basic image features, such as object color, texture, or orientation.

Although synthetic data are already widely used in the literature, there is still a great need for research in the analysis of the factors influencing the Reality Gap and the development of suitable methods in this respect. The question arises to how the Reality Gap can be reduced while at the same time keeping the modeling effort manageable. The aim is to optimize the process of modeling and data generation and to fully exploit the potential of synthetic sensor data. It is important in this context that not image differences and image properties influencing the human perception are used as a criterion but those relevant for the machine algorithm. Of special interest in the research of the Reality Gap is the consideration of content-equivalent real and synthetic image pairs. This aspect is also considered only rarely in previous publications, but it plays an important role to be able to evaluate the influence of the synthetic imaging parameters.

### 1.2. Objects of Investigation

To investigate these issues, vehicle detection on aerial imagery based on two-dimensional bounding boxes has been selected as an exemplary application in this paper. This is a current research area especially in UAV missions [9,33,34,35,36,37,38,39,40], furthermore, it forms the basis for more advanced applications such as object counting or tracking, and enables a direct performance comparison through appropriate metrics.

**Figure 1:**
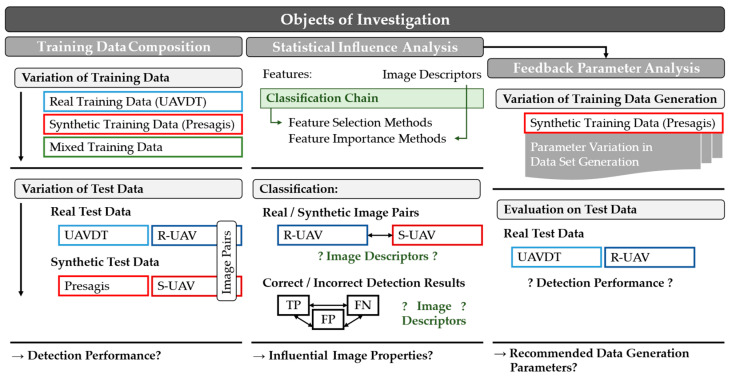
Schematic representation of the focus of investigation in this article, which includes the areas of training data composition, statistical influence analysis, and feedback parameter analysis in training data generation.

The literature research has shown that there is a clear need for research in the root cause analysis of the observed performance differences when using synthetic data and the subsequent identification of the image and simulation properties responsible for them. In the following, open research questions are therefore derived. The main objective is to develop a comprehensive and generally applicable research concept to find design guidelines for the generation and application of synthetic sensor data. Figure 1 shows the three components of this concept.

In the first block, the training data composition is investigated. Detection models are trained with different training datasets and evaluated on the corresponding test datasets and the real and synthetic image pairs. The associated research question is:

What performance differences do different training configurations (real, synthetic, mixed) show on the associated test data compared to independent content-equivalent image pairs? What conclusions can be derived with respect to the Reality Gap?

The objective in this first step is to evaluate the existing performance differences for a specific use case under defined boundary conditions and with a defined and comprehensive test database. It is claimed that a complete evaluation of the performance differences and their causes can only be conducted by additionally looking at image pairs, which is often neglected in previous publications.

The second block uses a classification chain with image descriptors as features to identify influential image properties. The associated research question is:

Which image properties play a role in the distinction between real and synthetic image pairs and between correct and incorrect detections? Which influencing factors can be derived to minimize the image and performance differences when using synthetic data?

The objective here is to analyze the Reality Gap statistically and independently of human perception. In contrast to the otherwise usual simple performance evaluation, causes for the observed image and performance differences are specifically determined here.

Finally, in the third block, the results of the two previous studies will be considered in the process of synthetic data generation, and optimization approaches will be investigated. The associated research question is:

What design parameters in synthetic data set generation positively and negatively affect model performance?

This approach aims at optimizing the synthetic training data generation. Not only the absolute performance is considered, but also the stability and the general applicability of the resulting models.

Thus, the overall objective of the article is to develop and exemplarily evaluate an overall concept that remains independent of the specific application and is generally applicable for identifying relevant influencing factors and deriving design guidelines when using any trainable deep learning based detector with synthetic data.

### 1.3. Content Overview

The first chapter presented the motivation, the state of the art, and the derivation of the research questions. Chapter 2 now describes the materials and methods needed to implement the research concept. This includes the selection of a real benchmark training dataset, the generation process of a synthetic training dataset, and the execution of real UAV flights to create content-equivalent real and synthetic image pairs. In addition, a test algorithm is selected and the developed statistical evaluation methodology is described. Finally, in Chapter 3, the description of the results follows. This includes the investigations on the training data composition, the identification of influential image features from the statistical evaluation methodology, and the optimization of the synthetic training data generation by evaluating different parameter variations. Finally, the main results are summarized in tabular form in Section 3.4 and can be considered as design guidelines for the use of synthetic sensor data. In the discussion in Chapter 4, the results are compared with previous findings from the literature. Chapter 5 provides a conclusion and an outlook on future and further research activities.

## 2. Materials and Methods

The datasets used are now presented. Since no generally applicable detection models are available for the described use case of UAV based vehicle detection, real and synthetic training and test datasets are necessary for learning the corresponding models. For a detailed evaluation, coupled real and synthetic image pairs are also used in this paper. The generation process and the necessary synthetic remodeling are also described.

Furthermore, the selection of the detection algorithm is discussed and the basics of the statistical evaluation procedure used in the second part of the investigations are described. This includes the selection of the image descriptors and the configuration of the classification chain.

### 2.1. Real Training Dataset

**Figure 2:**
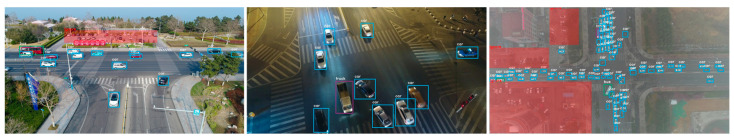
Annotated example images from the UAVDT dataset [41] showing some of the variations included. The areas marked in red were not annotated and are therefore not considered in the evaluation.

For effective training of the detector, the dataset must contain a sufficiently large amount of annotated images and also have a wide variation in terms of the size distribution of the objects to be detected, the perspective, the object orientation, and last but not least, the background and environmental conditions [39] (Figure 2).

In study [42], an overview of publicly available datasets with UAV aerial images can be found. Based on this, the UAVDT dataset [41] was selected as the real training dataset for the investigations. According to study [38], it is among the most challenging and largest drone based datasets. The dataset contains 40,000 annotated images with about 750,000 bounding boxes, three object classes, and a high variation in terms of flight altitude, viewing angles, objects, and especially, environmental conditions. Thus, all requirements are fulfilled. Figure 2 shows some sample images from the dataset.

### 2.2. Synthetic Training Dataset

In order to investigate the Reality Gap and the synthetic training behavior, a suitable synthetic training dataset is also required. Therefore, the synthetic simulation environment, the modeling, and finally, the process of dataset generation will be described.

#### 2.2.1. Simulation Environment and Modeling

For the synthetic data generation, a physically based visual sensor simulation is to be used, which supports a realistic representation and simulation of different ambient and environmental conditions. To increase the variation, the possibility of overlaying the rendered image with adjustable sensor effects is desired. A programming interface should enable automated data generation and annotation. For the investigations presented here, the Presagis Modelling and Simulation Suite [43] is used. It fulfills the required framework conditions, is specifically designed for real-time airborne simulations, and offers a module based tool chain with separate programs for modeling, terrain generation, and visualization.

With this simulation environment, the terrain of the University of the Bundeswehr Munich with the adjacent test flight area can be realistically modeled in the Common Database (CDB) [44] format. For this purpose, elevation data with a grid size of 1 m are overlaid with a georeferenced aerial photograph with a resolution of 20 cm per pixel. The geodata originate from the Bavarian Survey Administration [45]. A material classification is used for ground-level images to improve the level of detail by overlaying the aerial image with fine semi-transparent structures. In the last step, 3D models are added to the virtual world based on vector data. The existing buildings on the site were individually remodeled. The vegetation is simulated by 3D volume trees from SpeedTree [46], which also allow a simulation of seasons by different model versions. Moreover, a dataset of 38 different 3D vehicle models is used for the detection task [47], which were additionally recolored according to the global color distribution for cars, finally resulting in 80 used models [48]. The vehicle models are dynamically placed at runtime.

#### 2.2.2. Training Data Generation and Parameter Distribution

Based on this modeling, a synthetic dataset is now generated via the programming interface of the Presagis simulation environment, which can thus be used for training a detector model with synthetic data. The implementation was carried out in C++.

**Figure 3:**
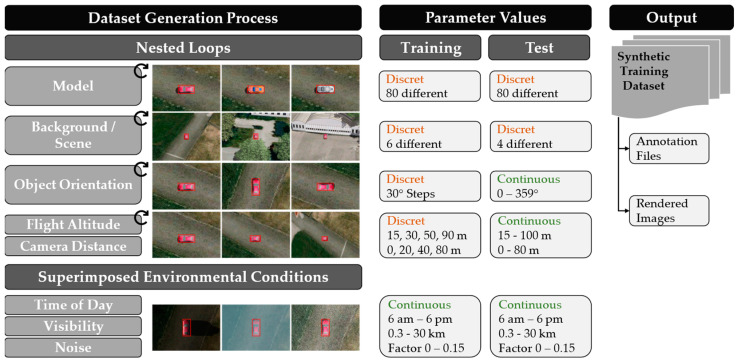
Schematic representation of the synthetic training data generation process. The effects and the discrete or continuous gradations of the parameter values are shown. The result is a synthetic training dataset with rendered images and the respective annotation files.

Figure 3 shows the generation process. In the first step, nested loops iterate over the vehicle models, scenery, object orientation, flight altitude, and camera radius. Then, the images are overlaid with random values for the time of day, visibility, and noise parameters to further increase variation. In order to be able to investigate individual parameter influences, the dataset initially contains only one vehicle per image, whose position varies randomly within the image section.

Figure 3 also shows the parameter gradations used in the generation. It should be emphasized that these are mostly discretely distributed in the training data, but continuous as far as possible in the associated test data. In this way, it could be additionally investigated whether a model trained with discrete gradations is able to generalize between all continuous gradations occurring in reality and whether thus the step size for the training data generation was chosen appropriately.

The result is a dataset with over 93,000 rendered images and the annotation files associated with each. In accordance with the UAVDT dataset, the images have a resolution of 1024  ×  540 pixels.

**Figure 4:**
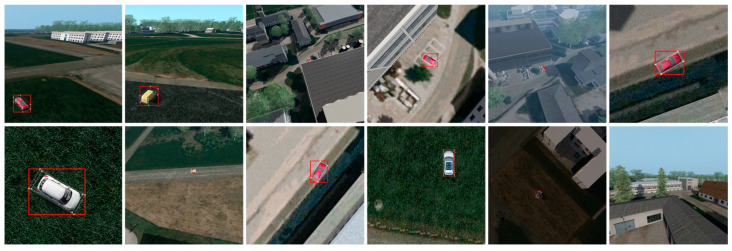
Annotated example images from the synthetically generated training dataset, which are also intended to illustrate the included variations.

Figure 4 shows some example images from the synthetic training dataset. Overall, the presented methodology allows to generate fully annotated image data with variable parameters and is transferable to any simulation environment with an appropriate programming interface.

#### 2.2.3. Parameter Variation in Dataset Generation

**Figure 5:**
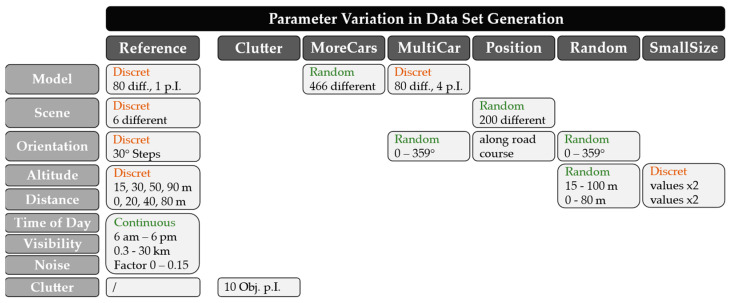
Overview of parameter variations in synthetic training data generation. p.I.: per Image; Obj.: Objects.

The third part of the research questions presented in Section 1.2 deals with the optimization of synthetic training datasets. For this purpose, different parameters of the dataset generation have to be varied in a decoupled way in order to evaluate their influence on the performance of the models. Figure 5 shows the six different variations with the corresponding parameter values. All datasets contain the same number of training images to exclude dependencies.

The synthetic dataset described in Section 2.2.2 serves as a reference and contains mostly discrete parameter distributions. In order to increase the proportion of clutter objects in the image and to investigate the resulting effects, ten additional objects are placed at different distances and orientations around the vehicle model in the “Clutter” dataset under otherwise identical conditions. In the “MoreCars” dataset, the variance in this parameter is increased by using 466 instead of only 80 different 3D vehicle models. The reference dataset always contains only one vehicle per image, whose position varies by random cropping of the image. In the “MultiCar” dataset, however, four vehicles are randomly placed in the camera’s field of view instead. As a result, these also exhibit a randomly distributed object orientation. Instead of six fixed vehicle locations, the “Position” dataset randomly switches between 200 different locations, with 150 positions distributed across different road types and 50 positions across different terrain types. These thus cover a broad scenic variety. This also has the advantage that the vehicles in this case are aligned along the road course, which according to study [49], is crucial for generating realistic scenes and reducing the Reality Gap. In the “Random” dataset, the geometric object parameters such as flight altitude, vehicle orientation or distance to the object are not varied in discrete steps but are continuously distributed between certain threshold values similar to the test data generation. The last dataset differs from the reference only with respect to the object sizes, since flight heights and object distances are used that are larger by a factor of two.

### 2.3. Real and Synthetic Image Pairs

For a detailed analysis, an evaluation of the image differences and performance differences between content-equivalent real and synthetic image pairs is essential. The dataset generation is based on the systematic execution of real UAV flights for data acquisition and the replication of synthetic duplicates based on the also recorded telemetry data.

#### 2.3.1. Real Data Acquisition

**Figure 6:**
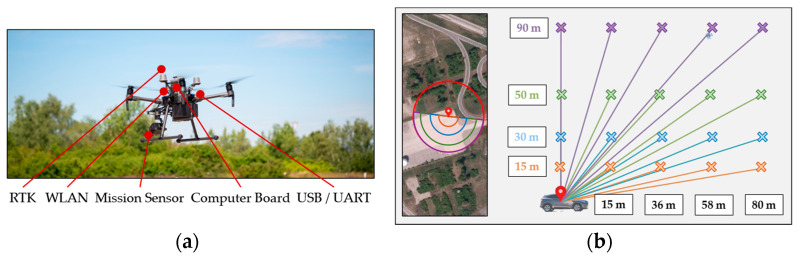
Real flight setup for data acquisition: (**a**) Hardware configuration of the multicopter system used with the corresponding extensions; (**b**) Semi-circular flight pattern to capture the different object views. Different gradations of flight altitude and distance to the object also generate a variation in relation to the angle of view.

A DJI Matrice M210 RTK V2 quadrocopter with Zenmuse XT2 sensor system and external Nvidia Jetson TX2 computer board is used for data collection. The acquisition of highly accurate position data with the integrated Real-Time Kinetics (RTK) is of particular importance, as these data form the basis for the generation of the synthetic duplicates in the second step. A fully automated flight execution allows reproducible data acquisition under different environmental conditions. Figure 6a shows the multicopter with hardware setup.

Four locations with different surfaces and background scenarios are selected at the test flight area of the University of the Bundeswehr. The test vehicles are statically positioned. Three vehicle types with different characteristics were used (van, SUV, small car). A semicircular flight pattern around the vehicle is used to capture all object orientations in equal numbers (see Figure 6b). A sensor image is captured in 20° steps. The discrete horizontal and vertical steps are also shown in Figure 6b and are used to capture the different viewing angles.

**Figure 7:**
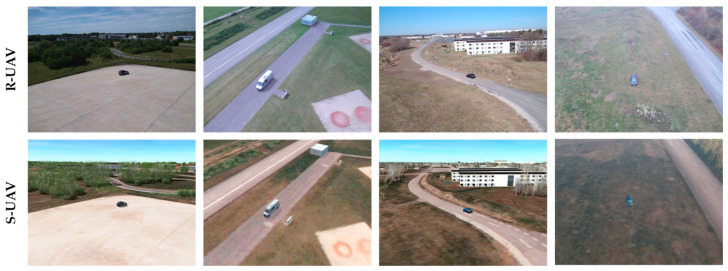
Comparison between real sensor images (R-UAV) and the corresponding synthetic duplicates (S-UAV) for the four acquisition positions.

In 91 flight missions, 4516 aerial images were generated in the aforementioned way. Due to the automated flight execution and the partly decoupled acquisition of different parameters, images could be generated which differ only with regard to one parameter, e.g., vehicle type, scenery, background, season or cloud coverage. This allows a very detailed evaluation. The first row of Figure 7 shows sample images of the flown dataset, which will be referred to as R-UAV. For each image, an annotation file is stored. This file contains the telemetry data, context parameters, and the manually labeled bounding boxes.

#### 2.3.2. Synthetic Duplication

The modeled virtual world also includes the test flight area used for the real flights. The modeling process is described in detail in Section 2.2.1. Based on the recorded telemetry data, a virtual camera is placed in the virtual world. Synthetic duplicates of the real images are rendered by taking into account sensor-related properties (e.g., resolution or field of view (FOV)) and simulating the corresponding environmental conditions (e.g., season, cloudiness, shadow cast). Figure 7 shows a comparison of such content-equivalent image pairs. Similar to the synthetic training data generation, the annotations are generated automatically.

### 2.4. Test Algorithm Selection

As already stated, UAV based vehicle detection has been defined as a use case for which a suitable test algorithm now has to be selected. The first classical detectors were mainly based on features such as colors, edges, or geometric constraints, are limited to simple scenarios, and are not robust to rotations or distortions [50,51,52,53,54,55]. Shallow learning based methods, on the other hand, already use more complex features and associated descriptors, which are then further processed by a classifier and used to assign regions of interest (ROI) [56,57,58]. For this purpose, the classifier has to be trained with the help of training data.

The newest and most powerful group of algorithms are deep learning based methods that use convolutional layers for feature extraction and classification. The disadvantage of these methods is the large amount of annotated data required, which leads to the training and test data problem. This is the motivation for using synthetic data. A distinction is made between two-stage and one-stage detectors. While two-stage detectors first generate possible ROIs and then classify them, one-stage methods combine both steps and are thus faster. For the selected use case, real-time online data processing on board the UAV is crucial. Since all sensor data processing takes place on embedded computer boards and yet a high frame rate is required, the focus here is on the use of one-stage methods. The YOLO detector is based on this concept and has been successfully used several times in the literature for the application considered here [9,37,39,40]. Therefore, and because it represents the properties of common algorithms very well, the YOLOv3 [17] detector was chosen as the test algorithm for the investigations carried out. Consideration of already available newer versions does not add any functional value to the experiments, but has the disadvantage of losing comparability with previous publications [42,59,60], which are based on similar datasets and thus allow an assessment of the stability of the presented evaluation concept. In addition, starting with version YOLOv3, a parallel further development of several research groups took place, whereby the subsequent versions are partially optimized for special objectives. Examples are anchor-free implementations, newer backbone frameworks, and approaches that are designed for simultaneous instance segmentation in addition to pure object detection. YOLOv5 also uses, for example, very comprehensive and far-reaching methods of data augmentation. All these advancements and the resulting more sophisticated network architecture generally increase detection performance. At the same time, however, the interpretability of the models and the identification of those influencing factors that are responsible for the Reality Gap become more difficult. Since this influence analysis is the main objective of the article, the presented investigation concept is evaluated in the first step with the established YOLOv3 detector. This is justifiable, since for these considerations, the absolute achieved detection performance plays a subordinate role and only relative performance differences are evaluated. Nevertheless, it should be noted at this point that the investigation concept can be applied to general deep learning based detectors. In future investigations, therefore, it can be shown on the basis of these basic results with the YOLOv3 network whether and to what extent the choice of other detector networks affects the identified influencing factors.

#### Implementation and Training of YOLOv3

Now some parameters will be listed, which are used for the training of the detector. For a description of how it works, please refer to study [17]. For each training dataset, nine anchor boxes are computed in advance by k-means clustering. The Darknet-53 network with 53 layers is used, which was pre-trained on the ImageNet dataset and has three different scaling layers. The training parameters used are largely consistent with the YOLOv3 configuration for the Pascal-VOC dataset. Each training phase starts with a warm-up phase of 1000 iterations. The input image size is 608  × 608 pixels, and the internal methods for data augmentation are also used. Based on the investigations in study [42], an exponential learning rate decay with exponent 0.9993 and initial learning rate of 0.004 is used, since this still provides stable results with a short training duration of 10,000 iterations. A Non-Maximum Suppression (NMS) with an Intersection over Union (IoU) threshold of 0.45 at the end of the network removes highly overlapping bounding boxes. Detections with a confidence value below 0.005 are not considered. A detection is also considered correct if its IoU value to ground truth is greater than 0.3. According to study [61], a human observer cannot distinguish this from an IoU threshold of 0.5, but the influence of annotation inaccuracies then becomes smaller.

### 2.5. Statistical Evaluation Method

The second part of the research deals with the identification of the influencing factors that are responsible for the Reality Gap and thus for the existing image differences and performance differences. A classification chain is applied, which uses image descriptor metrics as features. The goal is to establish a relationship between the target variable (domain of the input image or performance of the detection algorithm) and the independent variables (feature matrix with image descriptors) in order to derive the influential image features [59].

#### 2.5.1. Image Descriptors

Image descriptors provide a numerical description of various image properties. A distinction is made between metrics that describe more image content or more image quality, are based more on local or more on global calculations, and represent more human perception or more technical properties. In the selection, an attempt was made to consider all categories. In study [59], a detailed listing of the individual methods can be found. The following categories of image descriptors are used:

**MPEG-7 Color Descriptors** [62,63,64,65,66]. This group comprises four descriptors (SCD: Scalable Color Descriptor; CSD: Color Structure Descriptor; DCD: Dominant Color Descriptor; CLD: Color Layout Descriptor). The first two (SCD; CSD) describe the global and local color distribution in the image on the basis of a color histogram and thus capture not only the global color distribution but also the local spatial structure and arrangement of the colors. In contrast, the other two descriptors (DCD; CLD) describe only the dominant colors and their spatial distribution in the image.

**MPEG-7 Texture Descriptors** [62,63,64,65,67]. Textures describe visual patterns and have different properties that reflect their structural nature. One descriptor (EHD: Edge Histogram Descriptor) in this group captures the spatial distribution of edges in the image, the other (HTD: Homogeneous Texture Descriptor) is used to characterize repeating structures.

**Brightness/Luminance/Contrast** [68,69,70]. Several content and quality based methods for brightness and contrast calculation are listed in this group.

Color [69,71,72,73,74]. This group includes metrics that mainly look at color perception in terms of directly interpretable image properties, such as chromaticity, color cast, or color temperature.

**Image Quality** [70,75,76,77]. Here, methods are used that provide a technical or an aesthetic quality value or combine both. In some cases, interfering factors are also included and thus a measure of naturalness is calculated.

**Distortion/Blur/Noise** [69,78,79,80,81,82,83]. This group includes image descriptors for determining sharpness or blur in the image, as well as noise. They are all based on a technical calculation method.

**Figure 8:**
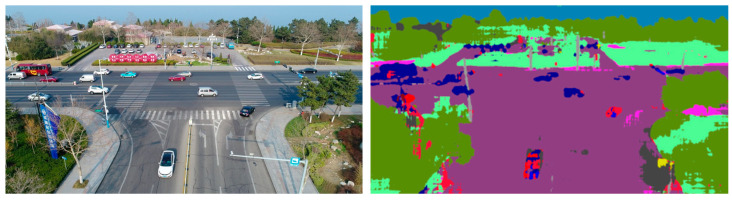
Visualization of the DeepLabv3 output image for visual verification of the segmentation results.

**Shape** [84,85]. This important group describes objects and shapes in the image by segmented binary masks and is thus representative of the image content. The segmentation masks are created in different ways:

- Foreground/background segmentation with Otsu threshold.
- Object/vehicle segmentation with DeepLabv3 network [86] trained on the COCO train2017 dataset.
- Semantic segmentation with DeepLabv3 network trained on the Cityscapes dataset. Figure 8 shows an example image.

**Environment** [87,88]. Neural networks are used here to predict environmental conditions in the image.

**Edges/Textures** [69,70,89]. Various features of the so-called Gray Level Co-Occurrence Matrix (GLCM), such as contrast, dissimilarity, homogeneity, are used to characterize textures. Further methods describe the number of edges in the image, and the smoothness as well as the spatial structure of the edge distribution.

#### 2.5.2. Classification Chain

**Figure 9:**
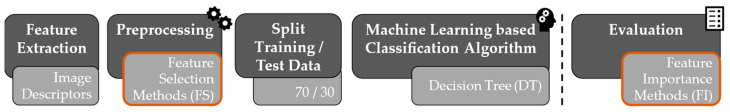
Schematic representation of the individual components of the classification chain used. The methods highlighted in orange are used to identify influential image features.

In this section, the structure and the components of the classification chain used for the statistical evaluation are described (see Figure 9). If the classification quality is high enough, Feature Selection (FS) and Feature Importance (FI) methods are used to identify those features or image properties that have a decisive influence on the model prediction.

The starting point is the feature extraction, which is based on the calculation of the image descriptors described in Section 2.5.1. Several preprocessing steps are used to complement and standardize the data, to balance the classes, and to reduce the correlation. Feature Selection methods are used to create a ranking of the most important image descriptors based on the data alone and independent of the classification algorithm used. A distinction is made between filter methods, wrapper methods, and embedded methods. In order to obtain reliable and stable results, methods from all three groups are considered. In total, the results of ten different FS methods are included in the evaluation. For a detailed list and description of the methods used, please refer to study [59]. Subsequently, the complete dataset is divided into training and test data in a ratio of 70 to 30. In the next step, the actual classification model is learned with the training data and optimized by model selection based on cross-validation. The decision tree algorithm is used as a classifier, since it provides an interpretable white-box model, is suitable for multi-class classification, and can also capture non-linear patterns. In the last block, the classification performance of the model is evaluated with the test data. In addition to the confusion matrix, the F1-score with micro-averaging is mainly considered. Moreover, Feature Importance methods are used here to rank the relevant features again, but in this case, based on the interpretation of the trained classification model. The objective is to highlight those features that make a decisive contribution to the explanation of the target variables. A distinction is made between model-specific and model-agnostic methods, the former being customized to a specific algorithm type, while the latter can be applied to any algorithm. A total of six different methods from both groups are used, which in turn are described in detail in study [59].

If a sufficiently high classification quality could be achieved with the described classification chain for the respective experiment considered, a stable and meaningful identification of the important image descriptors should be possible by comparing and combining the features selected by FS and FI methods. In the last step, these can be used to directly infer image properties that cause the Reality Gap and lead to the existing image and performance differences.

## 3. Results

In this article, several aspects are considered that play a role in the use of synthetic data. The investigations are therefore divided into three parts according to the research questions described in Section 1.2, which are analyzed in more detail with the experiments described below. In contrast to studies [42,59,60], all sub-experiments are based on the same datasets and models, which allows a general comparability for the first time. Furthermore, in the last part, additional feedback of these findings into the process of dataset generation are considered.

### 3.1. Examination and Evaluation of Training Datasets

In the first part of the investigations, the detector model is trained with three different training data configurations suitable for the use case of UAV based vehicle detection (see Figure 1, left). This includes the real UAVDT benchmark training data, the training data synthetically generated with the Presagis Modelling and Simulation Suite, and a mixed training dataset with the data from both domains, corresponding to a total synthetic proportion of 79%. These three models are then evaluated not only on the associated test datasets, but also on the content-equivalent real and synthetic image pairs, allowing detailed inferences about the Reality Gap to be derived. This enables a performance comparison between data from different domains as well as between different datasets of the same domain.

**Figure 10:**
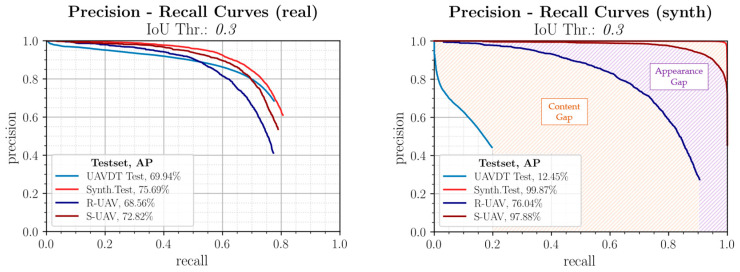

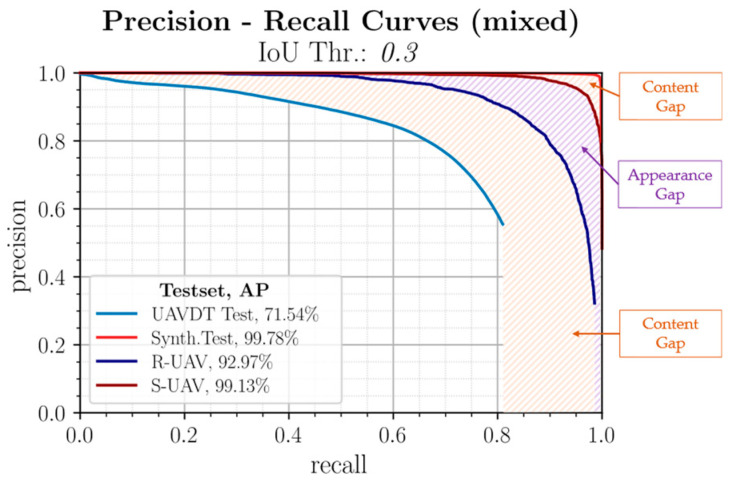
PR curves comparing the detection performances for the training configurations (real, synthetic, mixed) on the associated test datasets (UAVDT, Synth. Test) and the content-equivalent real and synthetic image pairs (R-UAV/S-UAV). The corresponding Average Precision (AP) is given in the legend.

Figure 10 shows the evaluation using Precision-Recall (PR) curves [60]. The area under the curve corresponds to the Average Precision (AP), which is used as a performance metric and should be as high as possible.

#### 3.1.1. Real Trained Model

The top left graph shows the evaluation of the model trained with the real UAVDT training data. This performs similarly well on all datasets and in both domains. It can be concluded that the real benchmark training data contains diversified features and thus the Reality Gap in this direction is small. When comparing performance between the test datasets, it is clear that higher performance is generally achieved on the synthetic test data because the features are more pronounced and the perturbation effects are smaller. In contrast, on unknown real data with partly different sceneries, the detection performance decreases, making this case decisive in the performance evaluation.

#### 3.1.2. Synthetic Trained Model

The purely synthetically trained model is shown in Figure 10, top right, and exhibits large differences in performance. It is obvious that this model shows an incipient overfitting to synthetic features due to the nearly ideal performance on the synthetic test data. For the real UAVDT test data, on the other hand, the model is not suitable due to different scenarios and object sizes, as it has a too low generalization capability for this and the Reality Gap in this direction is thus very large. In this context, it is interesting to note that on the R-UAV test images, which are also from the real domain, a good to very good detection performance with an AP of over 76% is achieved despite the synthetic training. Since the test flight domain was also used for data acquisition, this is most likely due to the similar sceneries and the similar object and context parameters. The synthetic duplicates, which are identical in content, show an even higher detection performance.

Overall, it can be shown that the very large Reality Gap is caused by two factors, whose influence is marked accordingly in Figure 10. The first part is based on differences in content between image data from the same domain and is also called Content Gap. Only the second part is actually based on differences in the image representation of image pairs with the same content and is therefore attributable to influences from synthetic image generation and is called Appearance Gap.

#### 3.1.3. Mixed Trained Model

The model trained with the mixed training data from both domains is shown in Figure 10 below. It achieves higher recall values, which indicates a better generalization ability and leads to the fact that this model achieves the highest detection performance among all training configurations. The ranking of the curves remains identical and again, albeit in a reduced form, the distribution of the Reality Gap can be seen. Of particular note is that the detection performance on the R-UAV dataset was very significantly increased by over 24 percentage points over the purely real benchmark training data by selectively adding synthetic training data with similar scenarios and conditions. This shows that the model can be specifically adapted to certain operating conditions by such training configurations with suitable synthetic data.

#### 3.1.4. Evaluation with Different IoU Thresholds

**Figure 11:**
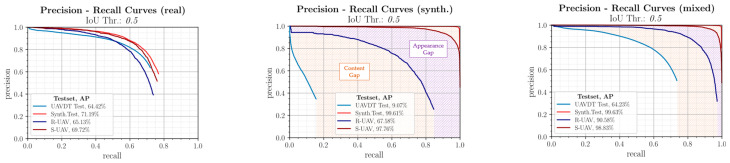
PR curves comparing detection performance for the different training configurations for an IoU threshold of 0.5.

Finally, it is evaluated how a change of the IoU threshold affects the described results. Figure 11 again shows the PR curves for the three training configurations considered, but with an IoU threshold of 0.5. As expected, the absolute detection performances decrease slightly. However, the focus of the investigations is on the performance comparison of the different configurations and the assessment of the correlations. Here it becomes clear that the results described in the previous sections can be confirmed. It can be seen that, on the one hand, the order of the curves remains identical and, on the other hand, a clear division into Appearance Gap and Content Gap can be seen. The evaluation is therefore stable to a change in the IoU threshold. Therefore, as before, an IoU threshold of 0.3 will be used as the basis for further investigations.

### 3.2. Statistical Influencing Factor Analysis

In the next part of the investigation, the factors responsible for the observed Reality Gap will be determined. Figure 1 shows, in the middle, schematically, the experimental setup. The classification chain described in Section 2.5 is used, which uses selected image descriptors as features and identifies those that have an influence on the detection result using FS and FI methods.

In the first step, the presented content-equivalent image pairs are analyzed to identify image features that lead to image differences between the domains and have to be considered in an optimized modeling and simulation. Based on this, the second part classifies between correct and incorrect detections based on the image information in the bounding box. This provides insights into the detection behavior of the black box detector model and corresponds to an analysis of the performance differences.

#### 3.2.1. Classification of Content-Equivalent Real and Synthetic Image Pairs

This part of the investigations is based on the real sensor images (R-UAV) and the synthetic duplicates with the same content (S-UAV). The image descriptor metrics computed on the two sets of data form the feature matrix for the binary classification into the target values “real” and “synthetic”.

The model calculated with the help of the classification chain described in Section 2.5.2 delivers a decision tree, which classifies the respective domain based on the values of certain features. The associated decision tree has a tree depth of four. The model thus has a medium complexity, which indicates a good agreement between real images and synthetic data. Nevertheless, an almost error-free classification quality with an F1 score of 0.996 is achieved on the unknown test data.

The successful classification now allows the evaluation of the most influential image descriptors by the total of 16 implemented FS and FI methods. If an image descriptor is evaluated as influential by less than three methods at the same time, it is not included in the evaluation.

Table 1 shows that noise has a disproportionately large influence, which suggests that the ideal rendered image data contain too little natural noise. In addition, several image descriptors are listed that represent different image properties in terms of color. This includes all four MPEG7 color descriptors, with the global dominant color in the image being numerically predominant. Furthermore, metrics for color cast and color temperature are listed, which is in accordance with the MPEG7 descriptors and also aims in the same direction. Although great importance was attached to the most exact possible content re-modeling, a feature from the semantic segmentation of the scenery is still included. However, this is most likely due to a combination of interferences or inaccuracies during the evaluation and is also at the back of the ranking.

In addition, Table 1 indicates for each listed image descriptor whether it represents global image properties describing the image in its entirety, or rather, those based on the distribution of local properties. It becomes clear that in the considered case, the global properties predominate. This is most likely due to the fact that similar local structures are present due to the detailed re-modeling, but the rendering in this case leads to global image differences.

The focus of the evaluation is explicitly not on human perception, but on the view of computer-aided image processing algorithms. Nevertheless, the identified influential image properties in this case can be visually reproduced directly on the basis of the example images from Figure 7. These contain too little noise or too few fine structures and details, especially in the area of the meadow and the road. Color differences are also clearly visible, which may well be perceived as color cast or differences in color temperature.

All in all, the aforementioned shows that with the presented method, an assignment of the image pairs to the respective domain is possible and that the image descriptors were chosen appropriately, since they describe the responsible image properties. In this way, several influencing factors were determined that affect the Appearance Gap and should be increasingly considered in future synthetic data generation. This forms the basis and prerequisite for the further analysis in the next section.

#### 3.2.2. Classification of Correct and Incorrect Detection Results

In the next step, performance differences will be analyzed to determine factors influencing the performance of deep learning based detectors. For this purpose, a grouping of the detections into TP (True Positive), FP (False Positive), and FN (False Negative) detections using the described classification chain are considered. The features for the data matrix are again the image descriptor values already described, but in this case, they are largely calculated based on the image information contained in the bounding box. In addition, the data matrix is extended by a semantic segmentation of the overall image and a list of context and object properties, such as object orientation, positioning, or flight altitude. The starting point for this analysis is the purely synthetically trained model, which is applied to the real R-UAV data. It achieves a very good AP of 76% due to the very high scenic similarity and the same geographic environment.

Despite the preference for a model that is as compact as possible in the course of model selection, the tree structure has 10 levels and is thus significantly more complex than in the differentiation between real and synthetic image pairs. Nevertheless, a very good classification quality with an F1 score of 0.968 can be achieved here as well. This again forms a good basis for the analysis of the influencing factors.

Table 2 again shows the ranking of the identified features. It is noticeable that there is less overlap between the features selected by FS and FI methods and thus overall fewer features are ranked as influential compared to the distinction between domains (see Section 3.2.1).

The descriptor for the dominant global colors in the image from the group of MPEG7 color descriptors is the most influential image descriptor in terms of both ranking and frequency. Whether the influence is primarily caused by the color of the background in the bounding box or by the color of the vehicle cannot be clearly distinguished. However, since the DCD descriptor was already numerically the most frequently occurring feature in the differentiation of the real and synthetic image pairs and since the same virtual modeling and simulation environment was used, a relevant proportion certainly originates from the background and can be assigned to the Appearance Gap in a certain way. The local distribution of dominant colors (CLD) also points in this direction.

In addition, the image acquisition position and thus the environment of the vehicle have an influence on the detection performance. Geometric recording parameters, object or environmental parameters such as vehicle orientation, flight altitude, angle of view, or time of year were also included in the feature matrix but had no influence on the detection result according to this evaluation.

The likewise listed measure for the aesthetic evaluation of the image quality cannot be assigned to any direct image property, but again primarily describes the Appearance Gap. The cause for the influence of the foreground segmentation and the contrast calculation from the GLCM matrix can also not be reliably derived. However, it is assumed that these parameters are related to the position and the vehicle environment, respectively.

The feature matrix also contains a semantic segmentation of the scenery. Features from this group were not found to be influential, because training and test data are from the same geographic environment and thus contain similar scenery. This highlights the plausibility of the analysis method.

In addition, disturbance factors, such as noise or blur, appear to have little or no influence on detection performance. The deep learning based detector is therefore relatively insensitive to such influencing factors or has already learned them to a sufficient degree during the training and can therefore generalize in this respect.

Table 2 shows that, here, too, the global image properties have by far the greater influence, which suggests that the overall impression of the image or the bounding box is much more important than the optimization of local parameters.

Overall, it can be stated that the presented methodology is thus also suitable for distinguishing correct and incorrect detections exclusively based on the image information in the bounding boxes and thereby achieves a very high classification quality.

### 3.3. Influence of Training Data Generation Parameters

In the third and last part of the investigations, an attempt is now made to optimize the training behavior and finally also, the subsequent detection performance by means of targeted parameter variation during training data generation. This closes the circle and now also includes the training as an elementary component of deep learning based detector networks in the analysis. Only purely synthetic training data are used in this evaluation, since only here a completely decoupled adaptation of the parameters for dataset generation is possible. The variations used were described in Section 2.2.3.

**Figure 12:**
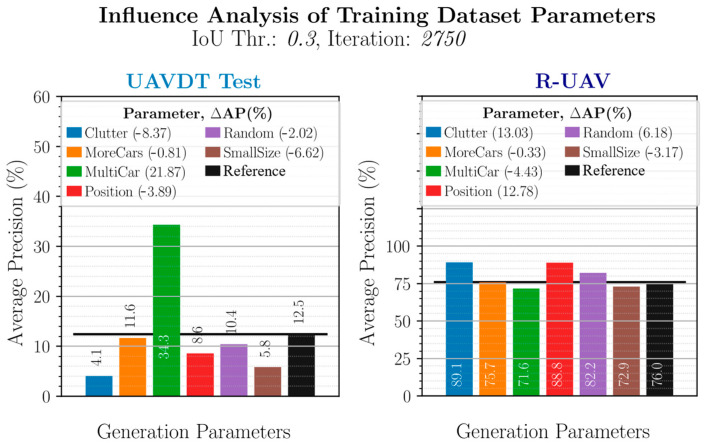
Comparison of the influence of different parameter variations in the synthetic training data generation on the performance of the detector model on the UAVDT and the R-UAV test data. In each case, the legend shows the change in AP compared to the reference model.

The models learned with the modified training data are evaluated for performance comparison on the real UAVDT and the real R-UAV test data. Figure 12 shows the results based on the AP obtained in each case for the different parameter variations and compares them with the AP of the reference model. In the legend, the respective differences are indicated numerically.

#### 3.3.1. Analysis on the UAVDT Test Data

Due to several unfavorable influences, but mainly due to the strongly varying scenery, the model learned with the synthetic reference training data achieves only a very low AP of 12.5% on the real UAVDT test data. The evaluation of other parameter variations should now show if and in which way the use of synthetic data can be optimized. The first variation (“Clutter”) tries to increase the robustness of the model during training by randomly placing clutter objects around the vehicle. However, the detection performance decreases noticeably. This is most likely since the UAVDT test data shows busy inner-city main roads where a vehicle is surrounded by other vehicles instead of clutter objects in most cases, and thus this training variation increases the Content Gap. In order to perform a decoupled influence analysis of different parameters, the reference training data always contains only one vehicle per image. By placing four additional vehicles in the image, the “MultiCar” training data increased the detection performance by almost 22 percentage points, as this now reduce the Content Gap. This is in agreement with previous findings and also shows the order of magnitude in which the Content Gap can affect detection performance.

Increasing the variation in the vehicle models used (“MoreCars”) did not result in any significant change. This suggests that 38 different 3D models, whose number is increased to 80 by recoloring, already cover the state space very well.

Using both a significantly larger number of positions (“Position”) and a continuous distribution of object parameters (“Random”) resulted in a model with lower detection performance. So did the use of smaller object sizes (“SmallSize”). This shows that the discrete parameter distribution can achieve better results than a continuous and random selection of the values and that the discrete step sizes were chosen appropriately when creating the reference training dataset. It has also been demonstrated that an increase in variance is not generally to be regarded as positive, as is usually assumed. It has been shown that a too high variance in individual parameters can even have a negative influence on the overall performance. It is assumed that the model then focuses too much on these parameters during training and the generalization ability decreases.

In addition to the Content Gap, for training data generation it is therefore important to ensure that the variance is as diversified as possible with parameters that are not disproportionately taken into account.

#### 3.3.2. Analysis on the R-UAV Test Data

Figure 12 also shows the analysis on the R-UAV test data. Due to the very high content and scene similarity between the synthetic reference training data and the real R-UAV data, the Content Gap is comparatively low and the reference model thus already achieves a very good AP of 76%. Additionally, the performance differences due to the different training data compositions are thus smaller.

Like the reference training data, the R-UAV data contains only one vehicle per image. This leads to the fact that now the reverse case is present as in the investigation of the UAVDT test data. This is also reflected in the performance differences. A placement of clutter now leads to an increase in performance, since in this case, it rather reduces the Content Gap and thus increases the model stability, whereas a placement of additional vehicles (“MultiCar”) has the opposite effect and leads to a slight decrease in performance.

Increasing the variation in vehicle positioning (“Position”) also has a positive effect in this case, since more of the later real deployment conditions and scenarios are already covered by the synthetic training data due to the same geographic environment. This also applies to the “Random” configuration, which also achieves better coverage of the test flight area with the synthetic data due to continuously distributed changes of the viewing angle. At this point, it should also be mentioned that the influence of the vehicle positioning coincides with the results of the classification analysis for the synthetic trained model on the real R-UAV data from Section 3.2.2 where the vehicle position was also evaluated as influential. This confirms the plausibility of the presented evaluation methodology.

The configurations “MoreCars” and “SmallSize” have no or only a minor influence on the detection result. This shows that the variation of the vehicle models is also covered by the model in this case and that the order of magnitude of the discrete parameter steps was chosen sensibly.

### 3.4. Summary and Derivation of Design Guidelines

In the following section, the most important findings of the investigations will now be summarized in tabular form. This should help to optimize the use of synthetic data and its benefits. In addition, the compilation also provides answers to the research questions posed in Section 1.2.

The first part of the investigations dealt with different training configurations (real, synthetic, mixed) and their effects on detection performance and the Reality Gap. Table 3 summarizes the results:

The second part considers a statistical evaluation based on a classification chain to identify influential image properties using image descriptors as features. A distinction is made between real and synthetic image data and between correct and incorrect detections. Table 4 collects the results:

In the third part, the focus is on the evaluation of different parameter variations in the synthetic training data generation. The goal is to optimize the use of synthetic sensor data by reducing the Reality Gap due to a suitable parameter distribution in the dataset. Table 5 collects the results:

## 4. Discussion

The results obtained by evaluating the research concept for the exemplary use case of UAV based vehicle detection are now compared with previous literature.

Real, synthetic, and mixed training datasets were considered and an optimization of the training data composition was performed. Consistent with the results presented in studies [25,28,32,90], training exclusively with synthetic data also resulted in lower performance than training with real data, in general. Nevertheless, again, the scenic similarity between training and test data has a very strong effect on this performance difference. In studies [18,91], it is concluded that the training value of a single simulated image is lower than that of a real image. Possible causes identified in this work include too little natural noise, varying coloration, and the generally lower level of detail. It was also shown in studies [28,90] that the best performance can be achieved with synthetic pre-training followed by fine-tuning on real data. Kar et al. [49] observed a similar division between Appearance Gap and Content Gap, and thus sought to optimize the choice of scene graph attributes and its composition during synthetic data generation. In contrast to the approach presented here, however, only the Content Gap is taken into account and no systematic automated evaluation procedure is used for influence analysis, but only subjective human evaluation based on sample images [49,91]. Nevertheless, the research presented here also confirmed the major impact of the Content Gap on detection performance. Furthermore, an additional evaluation was performed on real and synthetic image pairs with the same content. Only in this way is a complete evaluation of the performance differences possible.

Based on a classification chain with image descriptors as features, the factors influencing the Reality Gap were identified. It is important in this context that this type of evaluation is not based on human perception. Such causal analyses are rarely found in the literature. In study [32], the influence of certain image features on the detection performance is evaluated. However, this is only useful if the respective property can be separated directly. The Structured Domain Randomization (SDR) approach presented in study [92] does optimize the scene-based data composition. Nevertheless, since a neural network is used, deriving specific design guidelines in this case is difficult. The concept presented in study [93] also provides an evaluation concept, but uses different statistical methods and is only designed for image pairs and not for trainable deep learning based algorithms. The analysis concept considered in this paper was able to identify influential image properties for the described use case, which are responsible for the existing image and performance differences. In addition, the embedding in an overall concept is presented in this article in order to consider all aspects of the application.

In optimizing the synthetic data generation, not only the absolute performance but also the stability and general applicability of the models were evaluated. Again, there is little literature in this regard. It has been conjectured that a photorealistic representation in terms of human perception cannot be used as a benchmark for the quality of synthetic data [21,94,95]. This conjecture could be confirmed and it was shown that rather a suitable parameter distribution in the training data set and a minimization of the Content Gap is important. Furthermore, an increase of variance is not useful in general, but only if it is broadly diversified in content.

Through the presented experiments, this article has covered all areas that play a role in the use of synthetic data. The design guidelines established here and the insights gained can help to increase the benefits of using synthetic sensor data in the future.

## 5. Conclusions

When using deep learning based algorithms, the generation of suitable training and test data with synthetic simulation environments plays an important role. Using the example of airborne vehicle detection, this article focused on the boundary conditions and influencing factors to be considered, and provided design guidelines and recommendations for an optimized use of synthetic sensor data.

Thus, the overall objective was to develop and exemplarily evaluate an overall concept that remains independent of the specific application and is generally applicable for identifying relevant influencing factors and deriving design guidelines when using any trainable deep learning based detector with synthetic data.

Overall, the use of synthetic sensor data in deep learning based networks was analyzed comprehensively with content-equivalent real and synthetic image pairs, and it could be shown that the Reality Gap consists of a Content Gap and an Appearance Gap. The derived influential image properties and the suggestions for an optimized parameter distribution provide starting points for an effective application of synthetic data.

It remains to be verified how generally valid these design guidelines are and to what extent they depend on the current application and the currently used detector network. This applies, in particular, to detector networks that consider instance segmentation in addition to pure object detection. To increase the subsequent stability of the models, new approaches from the field of “Domain Adaptation” and “Adversarial Training” should also be taken into account in the synthetic training data generation in the future.

## Figures and Tables

**Table 1:** Ranking of the most influential image descriptors in distinguishing real and synthetic image pairs. The first line is a count of the number of those FS/FI methods that judged the image descriptor mentioned in the second line as influential. The last row states whether it is a global or local image descriptor. Dataset: R-UAV/S-UAV.

**No.**	11	9	9	8	7	6	5	4	4	4	4	3	3	3	3	3	3
**Image Descriptor**	Noise	Dominant Color (CLD)	Dominant Color (DCD)	Color Distribution (CSD)	Dominant Color (DCD)	Dominant Color (DCD)	Color Distribution (SCD)	Color Cast	Dominant Color (DCD)	Dominant Color (DCD)	Dominant Color (DCD)	Dominant Color (CLD)	Color Distribution (SCD)	Color Temperature	Segmentation Scenery	Color Distribution (CSD)	Color Distribution (CSD)
**G/L**	G	L	G	L	G	G	G	G	G	G	G	L	G	G	G	L	L

**Table 2:** Ranking of the most influential image descriptors in distinguishing correct from incorrect detections. Dataset: R-UAV test data; synthetically trained detector model. GLCM: Gray Level Co-Occurrence Matrix.

**No.**	13	9	7	7	6	5	5	4	3	3
**Image** **Descriptor**	Dominant Color (DCD)	Image Capture Position	Dominant Color (CLD)	Aesthetic Image Quality	Dominant Color (DCD)	Dominant Color (DCD)	Dominant Color (DCD)	Foreground Segmentation	Dominant Color (DCD)	GLCM: Contrast
**G/L**	G	G	L	G	G	G	G	L	G	G

**Table 3:** Evaluation results of different training data configurations.

**Real Benchmark Training Data**
- model achieves similar behavior on real and synthetic test data with a small Reality Gap in this direction.
- Unknown real image data with different scenery is the most complex form of evaluation.
- Publicly available real-world benchmark training data provides a general-purpose detector model with good performance.
**Purely Synthetic Training Data**
- The model has large performance differences and a large Reality Gap.
- If the real test data are from the same geographic environment and contains similar scenarios, good to very good detection performance is achieved.
- The Reality Gap consists of a Content Gap and an Appearance Gap.
- Purely synthetic training data are not sufficient to train a generally applicable detector model.
**Mixed Training Data**
- This model achieves the highest detection performance and generalization capability.
- This is the recommended training data configuration.
**The adaptability of the detector model to subsequent operational conditions can be significantly increased by selectively augmenting general real-world training data with specifically generated synthetic data from the subsequent operational area.**

**Table 4:** Results of the statistical evaluation procedure based on a classification chain.

**Classification of Content-Equivalent Image Pairs (Real/Synthetic)**
F1-Score: 0.996
- The synthetic data contain too little natural noise. - The synthetic modeling/virtual simulation environment leads to significant color differences, also in the form of color casts or different color temperatures.
- The identified image properties are visually comprehensible.
**Classification of Detection Results (TP/FP/FN)**
F1-Score: 0.968
- Color differences in relation to the dominant color of the background influence the detection result (Appearance Gap). - The image capture position and the vehicle surrounding also have an influence. - Geometric recording parameters, object or environmental parameters have no or very little influence, as well as interference effects.
**In general, global parameters predominate, so the overall impression of the image is more important than the optimization of local parameters.**

**Table 5:** Influences of different parameter variations in training data generation.

**Effects of Individual Parameter Variations**
- Adding clutter objects and increasing vehicle density has opposite effects depending on the test dataset. - Increasing the variation in image-capturing positions and using a continuous distribution of object parameters has opposite effects depending on the test dataset. - The variation of vehicles is very well covered by 38 3D models (80 models by recoloring).
**Conclusions**
- The Content Gap plays a decisive role here: The parameter distribution must be matched to the subsequent intended operation conditions. - A discrete parameter distribution provides a very good starting point and prevents overfitting the model to certain parameters. - An increase in variance is not to be considered positive in general, but only if it is diversified and individual parameters are not disproportionately taken into account.

## Data Availability

The data are not publicly available due to legal restrictions.
